# Lanthanide-Dependent Methanol Metabolism of a *Proteobacteria*-Dominated Community in a Light Lanthanide-Rich Deep Environment

**DOI:** 10.3390/ijms23073947

**Published:** 2022-04-01

**Authors:** Agnieszka Daszczyńska, Tomasz Krucoń, Robert Stasiuk, Marta Koblowska, Renata Matlakowska

**Affiliations:** 1 Department of Geomicrobiology, Institute of Microbiology, Faculty of Biology, University of Warsaw, 02-096 Warsaw, Poland; adaszczynska@biol.uw.edu.pl (A.D.); r.stasiuk@biol.uw.edu.pl (R.S.); 2 Department of Environmental Microbiology and Biotechnology, Institute of Microbiology, Faculty of Biology, University of Warsaw, 02-096 Warsaw, Poland; tkrucon@biol.uw.edu.pl; 3 Laboratory of Systems Biology, Faculty of Biology, University of Warsaw, 02-106 Warsaw, Poland; mk.koblowsk@uw.edu.pl; 4 Institute of Biochemistry and Biophysics, Polish Academy of Sciences, 02-106 Warsaw, Poland

**Keywords:** methanol, XoxF, lanthanides, black shale, metagenome, metaproteome

## Abstract

This study investigated the occurrence and diversity of proteobacterial XoxF-type methanol dehydrogenases (MDHs) in the microbial community that inhabits a fossil organic matter- and sedimentary lanthanide (Ln^3+^)-rich underground mine environment using a metagenomic and metaproteomic approach. A total of 8 XoxF-encoding genes (XoxF-EGs) and 14 protein sequences matching XoxF were identified. XoxF-type MDHs were produced by *Alpha*-, *Beta*-, and *Gammaproteobacteria* represented by the four orders *Methylococcales*, *Nitrosomonadales*, *Rhizobiales*, and *Xanthomonadales*. The highest number of XoxF-EG- and XoxF-matching protein sequences were affiliated with *Nitrosomonadales* and *Rhizobiales*, respectively. Among the identified XoxF-EGs, two belonged to the XoxF1 clade, five to the XoxF4 clade, and one to the XoxF5 clade, while seven of the identified XoxF proteins belonged to the XoxF1 clade, four to the XoxF4 clade, and three to the XoxF5 clade. Moreover, the accumulation of light lanthanides and the presence of methanol in the microbial mat were confirmed. This study is the first to show the occurrence of XoxF in the metagenome and metaproteome of a deep microbial community colonizing a fossil organic matter- and light lanthanide-rich sedimentary environment. The presented results broaden our knowledge of the ecology of XoxF-producing bacteria as well as of the distribution and diversity of these enzymes in the natural environment.

## 1. Introduction

Methanol is a common intermediate metabolite found in eukaryotes and prokaryotes [1]. In oceans, phytoplankton produces the highest amount of methanol [2], while in terrestrial ecosystems, methanol emission is mainly attributed to plants [3,4,5] and, to a lesser extent, to the decay of lignocellulosic plant material [6,7,8]. The presence of methanol in the environment has also been linked with methanotrophs, which oxidize methane.

Methanol formed from methane can be subsequently oxidized by methanotrophs to formaldehyde. It can also diffuse out from the periplasm of cells and be used by other nonmethanotrophic methylotrophs [9]. The bacterial oxidation of methanol to formaldehyde is mediated by enzymes known as pyrroloquinoline quinone-dependent methanol dehydrogenases (MDHs) which are soluble, periplasmic proteins [10,11]. So far, two types of MDHs have been described—MxaFI and XoxF. MxaFI, which has been known for more than 40 years, is a calcium-dependent heterotetramer [12,13,14,15]. In turn, XoxF, which was recently discovered, is a lanthanide (Ln^3+^)-dependent homodimer. The first reports on XoxF-type MDHs were published between the years 2012 and 2014 [16,17,18]. These MDHs bind preferably to Ln^3+^ of low atomic weight (from La to Eu) in their active site [19,20,21,22,23]. XoxF proteins were characterized to exhibit sequence divergence and phylogenetically classified into five major clades (XoxF1–XoxF5) by Chistoserdova in 2011 [24]. Later studies indicated that XoxF-like sequences are commonly found in various phylogenetically distant methanol-oxidizing bacteria and that more XoxF clades may possibly exist [17,18,25,26].

The present work is part of the research dedicated to microbiological transformations of fossil organic matter occurring in Lopingian Kupferschiefer black shale, which is located in the Fore-Sudetic Monocline (SW Poland). This study analyzed the processes of methanogenesis, as well as of methanotrophy and methylotrophy. The Kupferschiefer deposit is characterized by a very high content of fossil organic carbon (up to 30 wt %) of type II kerogen. Microbial transformations of fossil organic matter lead to the activation of fossil carbon and its inclusion in the global cycle. Moreover, the deposit has a significant concentration of Ln^3+^ and is especially enriched with light Ln^3+^ such as cerium, lanthanum, and neodymium [27,28]. In this paper, we focus on one of the stages of methanotrophy and methylotrophy, namely, methanol oxidation, with the use of XoxF-type MDHs in which light lanthanides play a crucial role.

**Figure 1:**
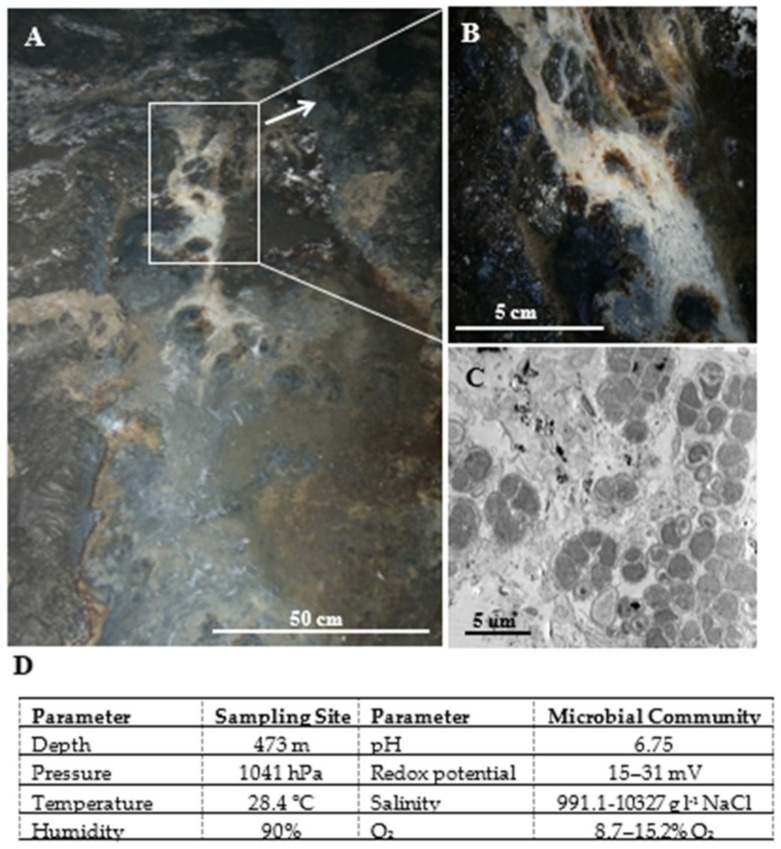
View of the microbial mat sampling site in Lubin mine (**A**). Enlarged view of the of microbial mat (**B**). Transmission electron microphotograph of an ultrathin section of the microbial mat (**C**). Physicochemical characteristics of the sampling site and the studied microbial community (**D**).

The aim of our study was to detect and characterize XoxF-type MDHs in the *Proteobacteria*-dominated microbial community that occurs in the form of a mat in the waters of an underground copper mine (SW, Poland) (Figure 1).

In this study, we examined the following: (1) the taxonomic composition of the studied microbial community, particularly, the *Proteobacteria* phylum, (2) the metagenome of the microbial community for the detection and characterization of XoxF-type MDH-encoding genes (XoxF-EGs), and (3) the metaproteome of the microbial community for the detection and characterization of XoxF-type MDH proteins. Additionally, we determined the content of light Ln^3+^ as well as checked the presence of methanol in the microbial community.

## 2. Results

### 2.1. Overall Results of Metagenomic and Metaproteomic Analyses

The metagenome of the studied microbial community was a 3.46 Gb data set of three samples that contained 25,136,545 reads with an average length of 134 nt. In total, 45,037 contings (>500 nt) were assembled. To estimate microbial richness at different taxonomic levels, rarefaction curves were generated (Figure S1). The rarefaction curves reached a plateau in the case of two samples and appeared to flatten out for the third sample, suggesting that sufficient sequencing saturation was achieved for taxonomic evaluation.

The functional analysis of the metagenome based on KEGG Orthology classification revealed 24,547 genes (18,799 within *Proteobacteria*) assigned to specific pathways. A total of 50,401 sequences (67% of all identified genes) contained predicted proteins with unknown function or below the assessed threshold for confidence.

The functional analysis of 3999 sequences of proteins was performed using GhostKOALA automatic annotation and KEGG mapping service. In total, 60.8% of the data was successfully annotated to specific pathways. A total number of 2324 protein sequences were assigned to *Proteobacteria*.

In the further characterization of the metagenome and metaproteome, we focused on sequences belonging to *Proteobacteria*, with particular emphasis on XoxF enzymes.

### 2.2. Taxonomic Composition of the Proteobacteria-Dominated Microbial Community

In the studied microbial community, bacteria were found to be dominant (97.89%), while archaea (0.50%) and fungi (1.55%) were less abundant.

**Figure 2:**
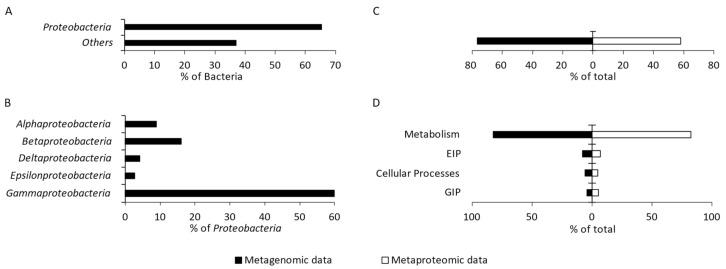
Percentage of *Proteobacteria* in the studied microbial community (**A**). Diversity and percentage of the identified proteobacterial classes (>1%) (**B**). Percentage abundance of proteobacterial KO (KEGG Orthologs) obtained from metagenomic and metaproteomic data among all identified bacterial KO (**C**). Number of proteobacterial KO representing four main functional categories; EIP—environmental information processing; GIP—genetic information processing (**D**).

A total of 51 bacterial phyla were identified, including 14 candidate phyla. *Proteobacteria* were determined as the predominant phylum, constituting 63.87% of the entire community (Figure 2A). The following bacterial phyla (each accounting for >1%) together constituted 23.91% of the community: *Bacteroidetes* (6.20%), *Firmicutes* (5.87%), *Actinobacteria* (3.43%), *Nitrospirae* (2.50%), and *Planctomycetes* (1.59%) (results not presented). The remaining 45 phyla (each accounting for <1%) constituted 16.54% of the studied community.

Within *Proteobacteria*, bacteria that belong to the class *Gammaproteobacteria* were found to be dominant (59.87%) (Figure 2B). In addition, the classes *Betaproteobacteria* (16.17%), *Alphaproteobacteria* (8.98%), *Deltaproteobacteria* (4.16%), and *Epsilonproteobacteria* (2.75%) were identified in this group. The remaining four identified classes together accounted for 0.34% of *Proteobacteria*, and 7.73% of *Proteobacteria* were not classified at all (Figure 2B).

### 2.3. Overall Characteristics of the Proteobacterial Metagenome and Metaproteome

The functional characteristics of the metagenome and metaproteome of the studied microbial community were created using GhostKOALA and KofamKOALA, which are the internal annotation tools of KEGG (Kyoto Encyclopedia of Genes and Genomes). The number of identified KEGG Orthologs (KOs) of proteobacterial origin was similar for the analysis of both metagenome and metaproteome. Of all the identified KOs, 76.59% and 58.11% were from the metagenome and metaproteome of *Proteobacteria*, respectively (Figure 2C). The following four major functional categories were distinguished: metabolism, environmental information processing, cellular processes, and genetic information processing. The proteins and genes from *Proteobacteria* responsible for metabolism predominated among the proteins and genes identified in the studied community, accounting for 82.32% and 82.51%, respectively (Figure 2D).

We detected both known types of MDHs (MxaFI-type and XoxF-type) in the studied microbial community. The analysis of the proteobacterial metagenome confirmed the presence of the following MDH-encoding genes: XoxF, Mdh1, Mdh2, MxaA, MxaC, MxaD, MxaG, MxaJ, MxaK, and MxaL. Moreover, proteobacterial metaproteome analysis allowed for the detection of XoxF, Mdh1, MxaF, Mdh2, MxaI, and MxaG sequences that matched the mentioned enzymes. In the further part of our work, we focused particularly on XoxF-type MDHs.

### 2.4. Detection and Characteristics of XoxF Produced by Proteobacteria

**Figure 3:**
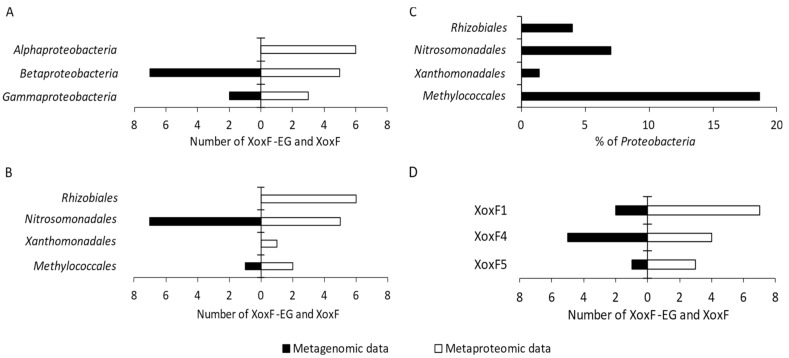
The number of XoxF-EG and XoxF enzymes detected in the proteobacterial metagenome and metaproteome among classes (**A**) and orders (**B**). Percentage distribution of bacterial orders with XoxF (**C**); number of the identified XoxF clades within *Proteobacteria* (**D**).

The metagenome and metaproteome analyses allowed identifying XoxF-type MDHs among *Betaproteobacteria* (seven XoxF-EGs and five XoxF), *Alphaproteobacteria* (no XoxF-EG and six XoxF), and *Gammaproteobacteria* (one XoxF-EG and three XoxF) (Figure 3A).

XoxF-EGs were detected in the metagenome of bacteria belonging to the orders *Nitrosomonadales* and *Methylococcales* within *Proteobacteria* (Figure 3B; Table 1). Additionally, XoxF enzymes were detected in the metaproteome of bacteria belonging to the orders *Rhizobiales*, *Nitrosomonadales*, *Xanthomonadales*, and *Methylococcales* (Figure 3B; Table 1). Most of the Ln^3+^-dependent MDHs were detected within the orders *Nitrosomonadales* (seven XoxF-EGs and five XoxF), *Rhizobiales* (no XoxF-EG and six XoxF), and *Methylococcales* (one XoxF-EG and two XoxF), while only one XoxF protein was found within *Xanthomonadales*.

The percentage of proteobacterial orders in the studied community, in which XoxF-EGs and/or XoxF enzymes were detected, is presented in Figure 3C. In total, 30.98% of proteobacterial taxa were detected with XoxF-EGs and XoxF enzymes. *Methylococcales* were the dominant order, constituting 18.63% of all *Proteobacteria*.

**Figure 4:**
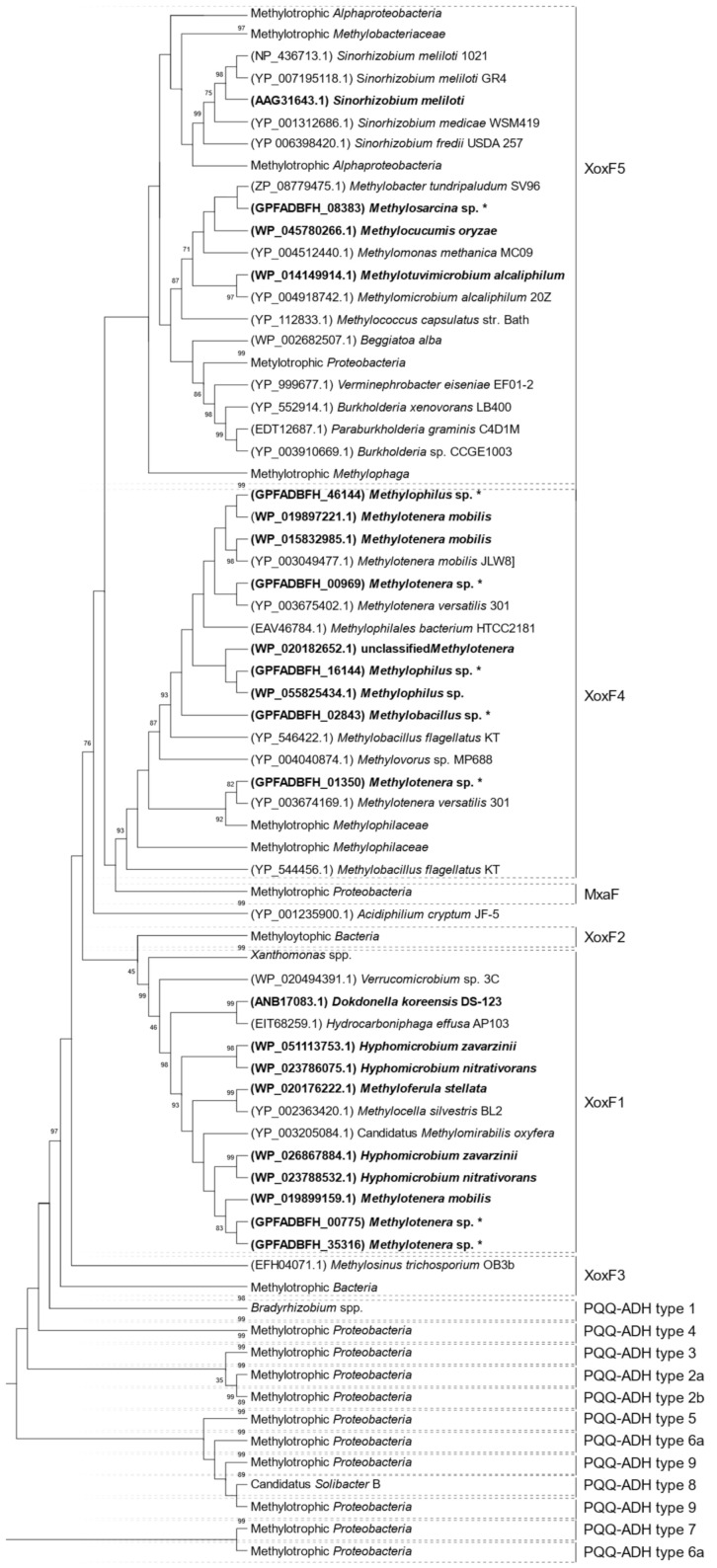
Compressed phylogenetic tree of alcohol dehydrogenases XoxF-MDHs, shown as a cladogram. Protein sequences detected in the biofilm are marked in bold (with/without an asterisk from the metagenome and proteome, respectively). Best hits from NCBI for metagenomics sequences: 1 WP_202051162.1; 2 WP_202051162.1; 3 WP_036300663.1; 4 WP_013149133.1; 5 WP_140003335.1 ; 6 WP_029147302.1; 7 WP_018987253.1; 8 WP_194741533.1; 9 WP_019899159.1. Bootstrap values above 75% are shown next to each branch. Compressed subtrees are described by the taxon of the lowest common ancestor. The tree with the highest log likelihood (−78,731, 80) is shown. The percentage of trees in which the associated taxa clustered together is shown next to the branches. The initial tree(s) for the heuristic search were obtained automatically by applying the Maximum Parsimony method. A discrete Gamma distribution was used to model evolutionary rate differences among sites (5 categories (+G, parameter = 13,551)). This analysis involved 253 amino acid sequences. In total, there were 533 positions in the final dataset.

Based on the metagenomic and metaproteomic data, the detected XoxF enzymes were divided into three clades using phylogenetic analysis, as follows: XoxF1, XoxF4, and XoxF5 (Figure 3D and Figure 4). The metagenomic data indicated XoxF4 as the dominant clade, while the metaproteomic data indicated XoxF1 as the dominant one (Figure 3D).

XoxF-EG belonging to the XoxF1 clade was detected in *Nitrosomonadales* (one XoxF1-EG); the most abundant XoxF4 clade was also detected within this order (five XoxF4-EGs). Clade XoxF5 was detected in *Methylococcales* (one XoxF5-EG). Proteins classified under the XoxF1 clade were most abundant within *Rhizobiales* (five XoxF1). The XoxF-type MDHs belonging to the XoxF1 clade were also detected in *Xanthomonadales* (one XoxF1) and *Nitrosomonadales* (one XoxF1). The XoxF4 clade was detected only within *Nitrosomonadales* (four XoxF4), whereas the XoxF5 clade was detected in two orders—*Methylococcales* (two XoxF5) and *Rhizobiales* (one XoxF5) (Table 1).

**Table 1:** XoxF-EG and XoxF enzymes (EC 1.1.2.10) detected in the studied microbial community.

Metagenomic Data					
NCBI Sequence No.		Score	TPM	Clade	Species/Genus
*Nitrosomonadales (Betaproteobacteria)*					
WP_194741533.1		1249	47.60	XoxF1	*Methylophilus* sp. 14
WP_019899159.1		157	5.10	XoxF1	*Methylotenera mobilis*
WP_018987253.1		1130	48.60	XoxF4	*Methylophilus methylotrophus*
WP_029147302.1		1081	38.08	XoxF4	*Methylophilus* sp. 5
WP_140003335.1		699	40.13	XoxF4	*Methylophilus medardicus*
WP_013149133.1		1181	41.39	XoxF4	*Methylotenera versatilis*
WP_036300663.1		304	51.04	XoxF4	*Methylotenera* sp. L2L1
*Methylococcales (Gammaproteobacteria)*					
WP_202051162.1		743	29.14	XoxF5	*Methylomicrobium* sp. RS1
		126	12.25	XoxF5	*Methylomicrobium* sp. RS1
**Metaproteomic data**					
**NCBI Sequence No.**	**Score**	**Seq(Sig)**	**emPAI**	**Clade**	**Species/Genus**
*Rhizobiales* (*Alphaproteobacteria*)					
AAG31643.1	56	1	0.05	xoxF5	*Sinorhizobium meliloti*
WP_020176222.1	211	4	0.20	xoxF1	*Methyloferula stellata*
WP_023786075.1	85	3	0.20	xoxF1	*Hyphomicrobium nitrativorans*
WP_023788532.1	74	2	0.14	xoxF1	*Hyphomicrobium nitrativorans*
WP_026867884.1	193	8	0.62	xoxF1	*Hyphomicrobium zavarzinii*
	236	9	0.72		
	135	2	0.15		
	194	4	0.26		
WP_051113753.1	141	5	0.36	xoxF1	*Hyphomicrobium zavarzinii*
*Nitrosomonadales (Betaproteobacteria)*					
WP_015832985.1	101	3	0.15	xoxF4	*Methylotenera mobilis*
WP_019897221.1	298	3	0.27	xoxF4	*Methylotenera mobilis*
WP_019899159.1	86	2	0.15	xoxF1	*Methylotenera mobilis*
	225	6	0.43		
	204	5	0.43		
	57	1	0,05		
	404	8	0.44		
	1195	14	1.73		
	405	8	0.58		
WP_020182652.1	77	1	0.05	xoxF4	*Methylotenera* sp. 1P/1
	199	1	0.10		
WP_055825434.1	64	1	0.06	xoxF4	*Methylophilus*
*Methylococcales (Gammaproteobacteria)*					
WP_014149914.1	74	1	0.05	xoxF5	*Methylomicrobium alcaliphilum*
WP_045780266.1	48	1	0.07	xoxF5	*Methylococcaceae bacterium* Sn10-6
	64	1	0.07		
*Xanthomonadales (Gammaproteobacteria)*					
ANB17083.1	129	3	0.21	xoxF1	*Dokdonella koreensis* DS-123
	158	4	0.29		

### 2.5. Detection of Light Ln^3+^ and Methanol in the Studied Microbial Mat

**Figure 5:**
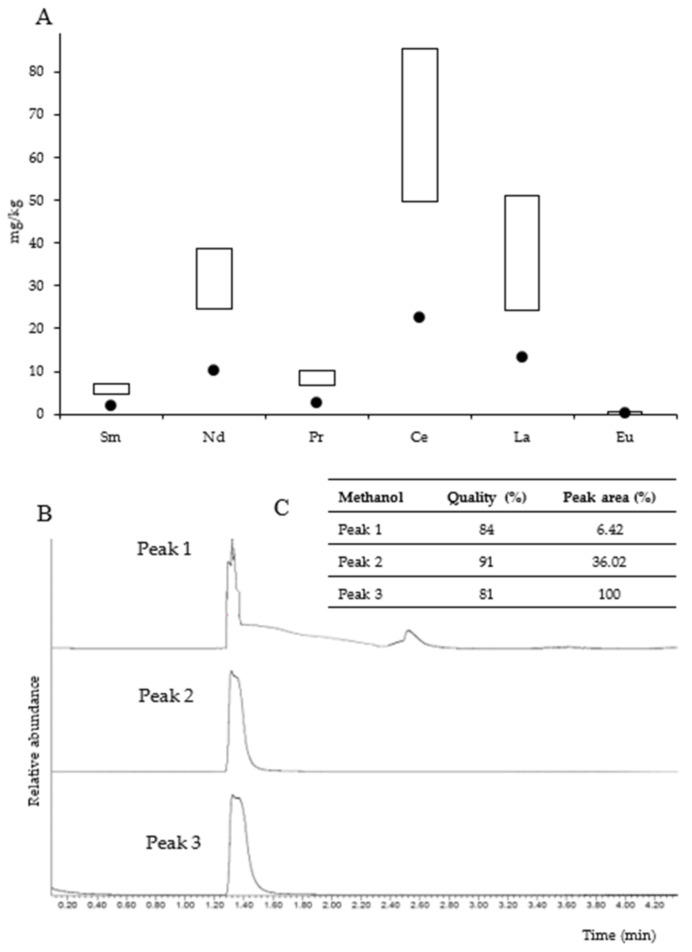
Content of light lanthanides in the microbial community (dots) and black shale (rectangles) (concentration ranges) (**A**). Detection of methanol in the microbial community—total ion gas chromatogram (fragments of chromatograms are presented without a unified scale of relative abundance axis) (**B**) and methanol identification (**C**).

Figure 5A shows the concentration of light Ln^3+^ determined in the Kupferschiefer black shale as well as in the studied microbial community. Due to the high heterogeneity of the black shale, Ln^3+^ concentrations are given in ranges in the figure. The total concentration of light Ln^3+^ in the black shale ranged from 110 to 194 mg/kg. The highest concentrations were found for cerium (49.6–85.5 mg/kg), lanthanum (24.2–51.2 mg/kg), and neodymium (24.6–38.6 mg/kg). On the other hand, europium was found at the lowest concentration (0.98–1.51 mg/kg).

The analyses revealed that the studied microbial mat was enriched with light Ln^3+^. The total concentration of light Ln^3+^ in the mat reached 50.71 mg/kg, which was lower compared to that of the black shale. The percentage of light Ln^3+^ in the microbial mat ranged from 25% to 41% of that in the black shale. Similar to the black shale, the microbial mat was identified to be particularly enriched with high concentrations of cerium, lanthanum, and neodymium (22.3, 13.3, and 10.2 mg/kg, respectively), whereas the concentrations of europium (0.41 mg/kg), samarium (2.04 mg/kg), and praseodymium (2.46 mg/kg) were found to be the lowest. Figure 5B,C presents the total ion chromatogram of the volatile organic compounds extracted from the samples of the microbial mat. Methanol (retention time, RT = 1.320 min) was identified in three samples, accounting for 6.42–100% (peak area) of all volatile organic compounds.

## 3. Discussion

Initially, Ln^3+^ were considered useless and biologically inert elements. However, the discovery of Ln^3+^-dependent MDHs shed light on their importance in microbial metabolism, which also indicated the potential role of bacteria in geochemical transformations on earth [18,29].

Currently, the bacterial utilization of Ln^3+^ is under intense investigation. At the same time, no research has been conducted in the natural environment, which is mainly rich in Ln^3+^, especially research that would both carry out the molecular characterization of XoxF and analyze the biogeochemical transformations occurring in the environment. Typically, studies focus on the isolation and characterization of a single species with a *xoxF* gene that can utilize methanol and/or on the characterization of expressed and purified XoxF enzymes [19,22,30,31,32]. A few reports are available from field studies. Two environmental research papers concerning Ln^3+^-dependent MDHs were published in 2015 describing the distribution of XoxF-containing methylotrophs in marine and freshwater surface waters [25,33]. In addition, Picone et al. (2020) [34] described the occurrence of XoxF-EGs in the microbial community of a volcanic soil based on a metagenomic analysis. Roszczenko-Jasińska et al. (2021) [35] conducted a field study on Ln^3+^ and detected XoxF proteins in the metaproteome of environmental lithobiontic bacterial community colonizing a light Ln^3+^-rich underground shale rock.

In this work, we tried to fill the knowledge gaps in the existing literature mainly by using environmental microbiology- and ecology-based methods and by addressing the issues relevant to these two fields. In particular, we aimed to highlight the characteristics of the studied underground environment that is rich in fossil organic carbon and sedimentary Ln^3+^. Our study material was a microbial community, not a single isolate, and the methodology used allowed for comprehensive research. We used a combination of geochemical analyses with high-throughput metagenomic and metaproteomic approaches. The results presented in this paper raise several noteworthy issues.

The study demonstrated the presence of methanol and Ln^3+^ in the analyzed microbial community (Figure 5), which might indicate three important facts: (1) bacteria (methanotrophs) present in the community oxidize methane and produce methanol; (2) the microbial community is capable of a methylotrophic lifestyle; (3) microorganisms can interact with Ln^3+^. Geochemical analyses were rather complementary to the methods used, yet they were crucial for investigating the Ln^3+^-dependent metabolism of methanol described in this work.

Among the *Proteobacteria* that dominated the community in the deep environment, we found some representatives of methane-oxidizing bacteria (Table S1), in which methanol is produced as the first intermediate during the oxygen-dependent oxidation of methane [17]. The group of genera detected in our study (relative abundance > 0.1%) included the aerobic methanotrophs listed by Semrau et al. in 2010 [36]: *Methylosarcina*, *Methylomonas*, *Methylobacter*, and *Methylomicrobium*. Additionally, in the metaproteome and metagenome of the studied community, we detected sequences that matched the enzyme responsible for methane oxidation—methane monooxygenase (EC:1.14.18.3), which supports the metabolic activity of the mentioned genera (Table S2). Moreover, the presence of methanotrophs was also confirmed by microscopic observations. Figure 1C shows the bacterial cells having a membrane structure typical of methanotrophs.

Another important issue is the presence of Ln^3+^ in the microbial mat. We did not detect Ln^3+^ in the underground water (data not presented). However, the analysis revealed that the content of Ln^3+^ in the microbial mat was higher compared to that in the black shale (Figure 5A). It is possible that bacteria producing XoxF were involved in the uptake of these metals. However, lanmodulin, which is the only lanthanophore known to be produced by bacteria to selectively bind and handle Ln^3+^ within the cell [37], was not detected in the studied microbial community. The significant accumulation of Ln^3+^ can be explained by their sorption by the compounds present in the extracellular matrix. This has been reviewed in detail by Andrès et al. (2003) [38] and described by Merroun et al. (2013) [39], Ozaki et al. (2006) [40], and Roszczenko-Jasińska et al. (2020) [41]. Furthermore, it was shown that the metabolism of nonmethylotrophic microorganisms such as *Bradyrhizobium* is stimulated by Ln^3+^ such as Ce, La, and Pr, resulting in the formation of extracellular polymeric substances [42]. It is worth mentioning that four Ln^3+^ (La, Ce, Pr, and Nd) have been identified as the most efficient in supporting microbial growth in comparison to Sm and Eu, which were found either to be less efficient or to not support the growth of microorganisms [19,21]. The selective uptake of these four Ln^3+^ into the cytoplasm was demonstrated by Ochsner et al. (2019) [42] and Mattocks et al. (2019) [43], and thus, the high concentration of these elements determined in the studied microbial community may indicate their selective uptake. Regardless of the mechanisms, the present study showed the enrichment of the microbial mat with Ln^3+^, which indicates the importance of bacteria, possibly including those capable of oxidizing methanol, in the redistribution of Ln^3+^ in the environment. However, we will investigate the uptake, trafficking, and utilization of Ln^3+^ in detail in the future, especially in the context of the acquisition of these elements from the black shale and the impact of bacteria on their redistribution in the environment.

In terms of the deep environmental ecology of microorganisms, the most significant finding of the present study is the taxonomic and metabolic diversity of XoxF-producing bacteria, including the phylogenetic characteristics of XoxF. Reports show that XoxF enzymes are widely distributed among *Proteobacteria* [17,24,25,44]. The ability to carry out Ln^3+^-dependent oxidation of methane is primarily specific to methanotrophic and methylotrophic bacteria. However, as recent studies indicate, XoxF enzymes are also found in various non-methylotrophic bacteria or species capable of methylotrophic co-metabolism [44]. In the studied community, we found XoxF-containing species that belong to obligatory methylotrophs among the orders *Methylococcales* and *Nitrosomonadales.* XoxF enzymes were also detected within *Rhizobiales*, which is an order of methanotrophs and non-methanotrophs. However, our study extends the existing knowledge, as the metaproteomic analysis indicated the presence of XoxF even in the order of non-methanotrophic *Xanthomonadales*, precisely within the aerobic heterotroph *Dokdonella koreensis* DS-123, described by Yoon et al. (2006) [45]. Only Chen et al. (2021) [46] reported that, apart from *Proteobacteria*, XoxF is encoded by the genome of the FCB (*Fibrobacteres*, *Chlorobi*, *and Bacteroidetes*) group, which is a superphylum.

Regarding the phylogenetics of XoxF, we found that the sequences of these enzymes are divergent [24]. The best known among the XoxF enzymes are the clades XoxF5 and XoxF4. XoxF5 enzymes are widespread among *Alpha*-, *Beta*-, and *Gammaproteobacteria*, while XoxF4 has been detected only in representatives of the family *Methylophilaceae* (*Betaproteobacteria*). In addition, the clades XoxF4 and XoxF5 appear to be mutually exclusive [47]. No biochemical data are available for XoxF3, and not much is known about XoxF1 and XoxF2 enzymes, whose distribution seems to be limited to specific taxa [17,24]. Our phylogenetic analyses revealed three clades, i.e., XoxF1, XoxF4, and XoxF5. Until now, in the field studies on the ecology of microbial communities concerning XoxF, either metaproteomic or metagenomic analysis has been used. Therefore, it should be emphasized that we exceptionally used both these analyses, which allowed comparing their results. It is worth noting that different clades were found dominant in each analysis (Figure 3D), and only one sequence was identified in both analyses—WP_019899159.1 (Table 1). Interestingly, we detected a relatively large number of sequences of XoxF1 (in particular, based on metaproteomic analyses), whereas studies of marine and terrestrial environments indicated that XoxF4 and XoxF5 are dominant [33,46,48,49,50].

In conclusion, our results contribute to an increased understanding of the ecology of XoxF-producing bacteria, as well as of the distribution and diversity of these enzymes in the natural environment. The obtained results indicate the potential ability of the studied microbial community to transform methanol using Ln^3+^-dependent XoxF. The results presented in this paper show that fossil sedimentary Ln^3+^ deposited in black shale may be used in bacterial metabolism and also highlight the influence of microorganisms on the redistribution of Ln^3+^ in the underground environment.

## 4. Materials and Methods

### 4.1. Site and Sample Description

The three samples of the microbial community analyzed in the study were aseptically collected from a depth of 473 m below sea level in the Lubin copper mine (SW Poland) at a temperature of 28.4 °C under 1041 hPa. The studied community occurred as an extensive microbial mat in the mine waters (Figure 1A,B). The characteristics of the sampling site and the studied microbial community are presented in Figure 1D. The pH of the collected samples was determined to be 6.75. The collected samples were stored at −80 °C or −4 °C, until they were subjected to laboratory procedures.

### 4.2. Isolation of DNA

DNA isolation from the three samples was performed according to the modified procedure of Zhou et al. (1996) [51]. Briefly, 10 g of sample was resuspended in 10 mL of DNA extraction buffer (0.1 M Na_2_EDTA, 0.1 M Tris–HCl, 0.1 M Na_2_HPO_4_, 1.5 M NaCl, and 1% hexadecyltrimethylammonium bromide; pH 8.0) containing proteinase K and lysozyme (75 µL; 10 mg/mL) and was incubated overnight at 37 °C under horizontal shaking. After incubation, 20% sodium dodecyl sulfate was added, and the samples were further incubated for 4 h at 65 °C. Then, the samples were centrifuged (6000× *g*, 10 min), and the harvested supernatants were mixed with an equal volume of a chloroform/isoamyl alcohol mixture (24:1) and centrifuged again (6000× *g*, 10 min). Next, the aqueous phases were collected and precipitated overnight with 0.6 volume of isopropanol at room temperature, and the samples were centrifuged once again (16,000× *g*, 20 min, 4 °C). After centrifugation, the obtained pellets were washed with 70% cold ethanol and allowed to dry. The DNA isolated from the pellets was resuspended in 50 µL of sterile deionized water and stored at −80 °C until analysis.

### 4.3. Sequencing and Analysis of DNA

A barcoded library was prepared from the isolated DNA using the Ion Xpress™ Plus Fragment Library Kit (Thermo Fisher, Waltham, MA, USA), according to the manufacturer’s instructions. The library was clonally amplified on Ion One Touch 2 system (Thermo Fisher) using the Ion PI™ Template OT2 200 Kit v2 (Thermo Fisher) and was sequenced on an Ion Proton sequencer using the Ion PI™ Sequencing 200 Kit v2 (Thermo Fisher), as per instructions. The reads were demultiplexed with Torrent Suite software.

The reads were subjected to qualitative preprocessing using the Trimmomatic tool (v. 0.38) [52] (SLIDINGWINDOW:4:15, HEADCROP:3, CROP:250, and MINLEN:35). The quality of the pre- and postprocessed reads was assessed using FastQC (v. 0.11.08). Human reads were removed using BMTagger (v 1.1.0) [53] and the human genome database GRCh38/hg38. Before taxonomic analysis, artificial duplicates were removed from the reads using the k-mer approach. The unassembled reads thus obtained were assigned to the taxa using Kraken2 (v. 2.0.8-beta) [54] and using prokaryotic, fungal, and viral protein sequences of the NCBI RefSeq database (ftp://ftp.ncbi.nlm.nih.gov/refseq/, accessed on 7 November 2019). Then, taxa abundances were averaged across the three samples. To evaluate the assembled contigs, QUAST (v. 5.0.2) [55] was used. Rarefaction curves for taxonomic analysis were generated using the krakefaction software (v. 0.2.0) (https://github.com/phac-nml/krakefaction, accessed on 30 January 2018) and plotted as the number of unique genera as a function of sequencing depth (Figure S1). Prior to functional analysis, the reads were merged and analyzed as a single sample. In the first step, the reads were assembled using SPAdes (v. 3.11.1) [56] with the “iontorrent” flag in the “careful” mode. Then, the assemblies were annotated using PROKKA (v. 1.13) [57] with default parameters and using KofamScan (v. 1.3.0) [58] with KOfam HMM profiles (v. 26.04.2021). The hits that had a bit score of <60 and an e-value of >1 × 10^-5^ were discarded. Based on the KO numbers and KEGG Orthology classification, the genes were assigned to metabolic pathways. The sequencing reads were realigned back to contigs using BBMap (v. 38.90) [59] with “vslow” parameter. Duplicates were removed using MarkDuplicates from the Picard tool kit. For all the samples, coverage, which was expressed as Transcripts Per Million [60], was normalized for each predicted gene and averaged. The taxonomy of each gene was determined using Kraken2. Ln^3+^ dehydrogenases were assigned to a given XoxF clade by phylogenetic analysis based on a phylogenetic tree [17] using the Maximum Likelihood method, Le Gascuel model, and 1000 bootstrap. Evolutionary analyses were conducted in MEGA X [61]. The accession numbers of the best hits were determined by aligning the XoxF protein sequences to the NCBI RefSeq database using BLASTp. Only the XoxF-EG sequences with unique best hits were taken into account, and duplicates were removed.

### 4.4. Isolation of Proteins

Proteins were extracted from the three samples as described by Ram et al. (2005) [62]. Briefly, 10 g of sample was resuspended in 100 mL of 20 mM Tris–HCl (pH 8), shaken for 3 min, and sonicated (Sonics Vibracell; LABOPLUS, ModelCV18head) on ice for up to 10 times for 1 min, with 1 min pauses in between. To remove the unlysed cells and cell membrane fragments, 100 mL of 0.4 M Na_2_CO_3_ (pH 11) was added to the sample, and the suspension was centrifuged (6000× *g*, 20 min, 4 °C). The collected supernatant was filtered through a 0.22 µm filter, and trichloroacetic acid was added to the solution at a ratio of 1:10 (*v*/*v*). The mixture was incubated overnight at 4 °C to allow for the precipitation of proteins and then centrifuged again (20,000× *g*, 10 min, 4 °C). After centrifugation, the aqueous phase was discarded, and the pellet containing the precipitated proteins was resuspended in 0.5 mL of prechilled methanol (4 °C) and centrifuged once again (20,000× *g*, 10 min, 4 °C). The resulting precipitate was dried and stored at −80 °C. The sample preparation was performed in triplicate.

### 4.5. Identification of Proteins

The isolated proteins were identified by liquid chromatography (LC) coupled to tandem mass spectrometry (MS/MS) using a nanoACQUITY (Waters) LC system and Orbitrap Velos mass spectrometer (Thermo Electron Corp., San Jose, CA, USA).

Identification was carried out at the Environmental Laboratory of Mass Spectrometry, Institute of Biophysics and Biochemistry (Polish Academy of Sciences, Warsaw, Poland). The equipment used was sponsored in part by the Centre for Preclinical Research and Technology (CePT), a project co-sponsored by the European Regional Development Fund and Innovative Economy, The National Cohesion Strategy of Poland.

Before the analysis, the proteins were subjected to standard “in-solution digestion”, which involved reduction with 50 mM Tris (2-carboxyethyl)phosphine (60 min, 60 °C), alkylation with 200 mM *S*-methyl methanethiosulfonate (45 min, room temperature), and overnight digestion with trypsin (Sequencing Grade Modified Trypsin; Promega V5111). After digestion, the resulting peptide mixture was applied to an RP-18 precolumn (nanoACQUITY Symmetry^®^ C18; Waters 186003514), along with water containing 0.1% trifluoroacetic acid used as the mobile phase. Subsequently, the peptide mixture was transferred to a nano-HPLC RP-18 column (nanoACQUITY BEH C18; Waters 186003545), using an acetonitrile (ACN) gradient (5–35% ACN in 180 min) with 0.05% formic acid as the mobile phase (flow rate 250 µL/min). The column outlet was directly coupled to the ion source of the spectrometer, which switched the mode from data-dependent MS to MS/MS. Each analysis was preceded by a blank run to avoid cross-contamination from previous samples.

The acquired raw data were processed by Mascot Distiller. This was followed by Mascot Search (Matrix Science, London, UK, onsite license) against the NCBI protein database. The peptides with a Mascot score exceeding a threshold value corresponding to <5% of the expectation value, which was calculated by the Mascot procedure, were considered as positively identified.

Metaproteomic analysis was performed using GhostKOALA automatic annotation and KEGG mapping service [63]. Before functional analysis, all replicates of the protein samples were merged and analyzed as a single sample. Duplicate sequences were deleted.

### 4.6. Ln^3+^ Concentration Analysis

Ln^3+^ concentration was estimated in the samples by ACME Analytical Laboratories Ltd. (Vancouver, BC, Canada; http://www.acmelab.com (accessed on 7 November 2019)). Inductively coupled plasma (ICP) atomic emission spectrometry or ICP–MS was used for this purpose. Standard ACME procedures were applied for sample preparation and analysis.

### 4.7. Extraction of Volatile Compounds

For extracting the volatile compounds, 5 g of the sample was placed in a test tube. The tube containing the sample was capped and placed at 100 °C for 12 h. Using a gastight syringe, gas evolving during sample heating was collected from the tube. The gaseous samples thus collected were analyzed using gas chromatography coupled to mass spectrometry (GC-MS).

### 4.8. Analysis of Volatile Compounds by GC–MS

Gaseous compounds were separated using an Agilent 7890A Series Gas Chromatograph interfaced to an Agilent 5973c Network Mass Selective Detector (Agilent Technologies, Santa Clara, CA, USA). A 5 cm^3^ gas sample was injected into an HP-1MS column (30 m × 0.25 mm I.D., 0.25 µm film thickness; Agilent Technologies, Santa Clara, CA, USA) with a splitless injector using helium as the carrier gas (1 mL/min). The ion source was maintained at a temperature of 250 °C. The GC oven was programmed with a temperature gradient starting at 35 °C (for 5 min) and gradually increasing to 100 °C at 12 °C/min.

MS analysis was carried out in the electron-impact mode at an ionizing potential of 70 eV. The mass spectra of the samples were recorded at the mass-to-charge (*m*/*z*) ratio of 0–150 (0–10.5 min).

Selected compounds were identified using Agilent Technologies Enhanced ChemStation (G1701EA ver. E. 02.00.493) and Wiley Registry of Mass Spectral Data (version 3.2; Palisade Corporation, 8th Edition with Structures (Copyright 1988–2000), and John Wiley and Sons, Inc. (Copyright 2000)) using a 3% cutoff threshold.

## Data Availability

The results of metagenomic DNA sequencing are deposited at: https://www.ncbi.nlm.nih.gov/bioproject/?term=+PRJNA776740.

## Supplementary Materials

The following supporting information can be downloaded at https://www.mdpi.com/article/10.3390/ijms23073947/s1.
