# Supplementary material for: Lanthanide-Dependent Methanol Metabolism of a Proteobacteria-Dominated Community in a Light Lanthanide-Rich Deep Environment
Source: Int J Mol Sci. 2022 Apr 1;23(7):3947. doi: 10.3390/ijms23073947 (PMC8999231; doi:10.3390/ijms23073947)
Supplement: Supplementary file 1 [file ijms-23-03947-s001.zip › ijms-1634001-supplementary.pdf]

Supplementary Materials

Lanthanide-dependent methanol metabolism of *Proteobacteria*-dominated community in light lanthanide-rich deep environment

Agnieszka Daszczyńska[1], Tomasz Krucoń[2], Robert Stasiuk[1], Renata Matlakowska[1*]

[1]Department of Geomicrobiology, Institute of Microbiology, Faculty of Biology, University of Warsaw;

[2]Department of Environmental Microbiology and Biotechnology, Institute of Microbiology, Faculty of Biology, University of Warsaw

Correspondence: r.matlakowska@uw.edu.pl

Table S1. Detected genera that Semrau et al. (2010) listed as aerobic methanotrophs.

Phylum	Family	Genus	avg(genus)	Species	reads ion21	reads ion22	reads ion23	% reads21	% reads22	% reads23	AVG	SD
Gammaproteobacteria	*Methylococcaceae*	*Methylobacter*	0.4085	*Candidatus* Methylobacter oryzae	1181	268	53	0.0546	0.0080	0.0335	0.0321	0.0233
				Methylobacter luteus	1974	348	62	0.0913	0.0105	0.0392	0.0470	0.0410
				Methylobacter marinus	390	49	16	0.0180	0.0015	0.0101	0.0099	0.0083
				Methylobacter sp. BBA5.1	5689	574	143	0.2631	0.0172	0.0904	0.1236	0.1263
				Methylobacter tundripaludum	4382	841	153	0.2027	0.0253	0.0967	0.1082	0.0893
				Methylobacter whittenburyi	222	36	7	0.0103	0.0011	0.0044	0.0053	0.0046
				unclassified *Methylobacter*	3434	726	106	0.1588	0.0218	0.0670	0.0825	0.0698
		Methylococcus	0.0113	*Methylococcus capsulatus*	305	111	26	0.0141	0.0033	0.0164	0.0113	0.0070
		Methylocaldum	0.0617	*Methylocaldum marinum*	607	146	21	0.0281	0.0044	0.0133	0.0152	0.0120
				Methylocaldum sp. 0917	60	13	5	0.0028	0.0004	0.0032	0.0021	0.0015
				Methylocaldum sp. 14B	67	22	4	0.0031	0.0007	0.0025	0.0021	0.0013
				Methylocaldum sp. BRCS4	129	11	6	0.0060	0.0003	0.0038	0.0034	0.0028
				Methylocaldum sp. SAD2	12	8	1	0.0006	0.0002	0.0006	0.0005	0.0002

Phylum	Family	Genus	avg(genus)	Species	reads ion21	reads ion22	reads ion23	% reads21	% reads22	% reads23	AVG	SD
				Methylocaldum szegediense	304	98	12	0.0141	0.0029	0.0076	0.0082	0.0056
				unclassified Methylocaldum	1029	260	56	0.0476	0.0078	0.0354	0.0303	0.0204
		Methylohalobius	0.0067	*Methylohalobius crimeensis*	227	86	11	0.0105	0.0026	0.0070	0.0067	0.0040
		Methylomicrobium	0.2300	*Methylomicrobium agile*	5450	929	176	0.2521	0.0279	0.1112	0.1304	0.1133
				Methylomicrobium alcaliphilum	184	56	13	0.0085	0.0017	0.0082	0.0061	0.0039
				Methylomicrobium buryatense	513	104	21	0.0237	0.0031	0.0133	0.0134	0.0103
				Methylomicrobium kenyense	696	141	35	0.0322	0.0042	0.0221	0.0195	0.0142
				Methylomicrobium sp. wino1	1386	237	36	0.0641	0.0071	0.0227	0.0313	0.0294
				unclassified *Methylomicrobium*	1117	274	44	0.0517	0.0082	0.0278	0.0292	0.0218
		Methylomonas	2.5868	*Methylomonas denitrificans*	2	2	0	0.0001	0.0001	0.0000	0.0001	0.0000
				Methylomonas koyamae	16454	4345	2711	0.7610	0.1305	1.7131	0.8682	0.7967
				Methylomonas lenta	1999	1048	148	0.0925	0.0315	0.0935	0.0725	0.0355
				Methylomonas methanica	10661	8269	902	0.4931	0.2484	0.5700	0.4371	0.1679
				Methylomonas sp. 11b	1201	284	59	0.0555	0.0085	0.0373	0.0338	0.0237
				Methylomonas sp. DH-1	231	76	28	0.0107	0.0023	0.0177	0.0102	0.0077
				Methylomonas sp. GJ1	1213	656	100	0.0561	0.0197	0.0632	0.0463	0.0233
				Methylomonas sp. Kb3	500	95	26	0.0231	0.0029	0.0164	0.0141	0.0103
				Methylomonas sp. LW13	268	68	22	0.0124	0.0020	0.0139	0.0094	0.0065
				Methylomonas sp. LWB	1643	277	98	0.0760	0.0083	0.0619	0.0487	0.0357
				Methylomonas sp. MK1	1754	371	73	0.0811	0.0111	0.0461	0.0461	0.0350
				unclassified *Methylomonas*	26204	8985	2403	1.2120	0.2699	1.5184	1.0001	0.6507
		Methylosoma	not found	n/a	n/a*	n/a	n/a	n/a	n/a	n/a	n/a	n/a
		Methylosarcina	5.7403	*Methylosarcina fibrata*	253415	39968	5971	11.7207	1.2005	3.7730	5.5648	5.4842
				Methylosarcina lacus	4752	828	132	0.2198	0.0249	0.0834	0.1094	0.1000
				unclassified *Methylosarcina*	3002	496	71	0.1388	0.0149	0.0449	0.0662	0.0647
		Methylosphaera	not found	n/a	n/a	n/a	n/a	n/a	n/a	n/a	n/a	n/a
		Methylothermus	not found	n/a	n/a	n/a	n/a	n/a	n/a	n/a	n/a	n/a
		Crenothrix	0.0435	*Crenothrix polyspora*	1592	396	71	0.0736	0.0119	0.0449	0.0435	0.0309

Phylum	Family	Genus	avg(genus)	Species	reads ion21	reads ion22	reads ion23	% reads21	% reads22	% reads23	AVG	SD
		Clonothrix	not found	n/a	n/a	n/a	n/a	n/a	n/a	n/a	n/a	n/a
Alphaproteobacteria	*Methylocystaceae*	*Methylosinus*	0.0083	*Methylosinus* sp. C49	12	7	1	0.0006	0.0002	0.0006	0.0005	0.0002
				Methylosinus sp. Ce-a6	16	23	1	0.0007	0.0007	0.0006	0.0007	0.0001
				Methylosinus sp. LW3	11	9	1	0.0005	0.0003	0.0006	0.0005	0.0002
				Methylosinus sp. LW4	10	13	2	0.0005	0.0004	0.0013	0.0007	0.0005
				Methylosinus sp. PW1	32	12	4	0.0015	0.0004	0.0025	0.0015	0.0011
				Methylosinus sp. R-45379	17	1	0	0.0008	0.0000	0.0000	0.0003	0.0004
				Methylosinus sporium	28	15	0	0.0013	0.0005	0.0000	0.0006	0.0007
				Methylosinus trichosporium	0	3	0	0.0000	0.0001	0.0000	0.0000	0.0001
				unclassified *Methylosinus*	85	64	8	0.0039	0.0019	0.0051	0.0036	0.0016
		Methylocystis	0.0447	*Methylocystis bryophila*	28	34	7	0.0013	0.0010	0.0044	0.0022	0.0019
				Methylocystis heyeri	53	38	4	0.0025	0.0011	0.0025	0.0020	0.0008
				Methylocystis hirsuta	59	41	6	0.0027	0.0012	0.0038	0.0026	0.0013
				Methylocystis parvus	84	81	7	0.0039	0.0024	0.0044	0.0036	0.0010
				Methylocystis rosea	85	112	6	0.0039	0.0034	0.0038	0.0037	0.0003
				Methylocystis sp. ATCC 49242	89	87	4	0.0041	0.0026	0.0025	0.0031	0.0009
				Methylocystis sp. B8	104	152	5	0.0048	0.0046	0.0032	0.0042	0.0009
				Methylocystis sp. MitZ-2018	22	33	6	0.0010	0.0010	0.0038	0.0019	0.0016
				Methylocystis sp. SB2	11	6	1	0.0005	0.0002	0.0006	0.0004	0.0002
				Methylocystis sp. SC2	26	49	2	0.0012	0.0015	0.0013	0.0013	0.0001
				unclassified *Methylocystis*	337	551	42	0.0156	0.0166	0.0265	0.0196	0.0061
	Beijerinckaceae	*Methylocapsa*	0.0073	*Methylocapsa acidiphila*	28	39	3	0.0013	0.0012	0.0019	0.0015	0.0004
				Methylocapsa aurea	40	36	5	0.0019	0.0011	0.0032	0.0020	0.0011
				Methylocapsa palsarum	41	26	1	0.0019	0.0008	0.0006	0.0011	0.0007
				Methylocapsa sp. S129	66	68	3	0.0031	0.0020	0.0019	0.0023	0.0006
				unclassified *Methylocapsa*	8	5	1	0.0004	0.0002	0.0006	0.0004	0.0002
		Methylocella	0.0062	*Methylocella silvestris*	105	82	10	0.0049	0.0025	0.0063	0.0045	0.0019
				Methylocella tundrae	29	53	2	0.0013	0.0016	0.0013	0.0014	0.0002
				unclassified *Methylocella*	2	1	1	0.0001	0.0000	0.0006	0.0003	0.0003
Verrucomicrobia	*Methylacidiphilaceae*	*Methylacidiphilum*	0.0078	*Methylacidiphilum fumariolicum*	10	9	3	0.0005	0.0003	0.0019	0.0009	0.0009

Phylum	Family	Genus	avg(genus)	Species	reads ion21	reads ion22	reads ion23	% reads21	% reads22	% reads23	AVG	SD
				Methylacidiphilum infernorum	22	35	5	0.0010	0.0011	0.0032	0.0017	0.0012
				Methylacidiphilum kamchatkense	10	5	0	0.0005	0.0002	0.0000	0.0002	0.0002
				Methylacidiphilum sp. Phi	30	22	3	0.0014	0.0007	0.0019	0.0013	0.0006
				Methylacidiphilum sp. Yel	13	6	0	0.0006	0.0002	0.0000	0.0003	0.0003
				unclassified *Methylacidiphilum*	85	81	6	0.0039	0.0024	0.0038	0.0034	0.0008

* n/a - not applicable

Table S2. Methane monooxygenase sequences detected in the metaproteome and metagenome of the studied microbial community.

NCBI sequence no.	Description	Genus/Species	Order (Class)
**Metagenomic data**			
WP_202053531	methane/ammonia monooxygenase subunit A [EC:1.14.18.3 1.14.99.39]	*Methylomicrobium* sp. RS1	*Methylococcales (Gammaproteobacteria)*
WP_020564882	methane/ammonia monooxygenase subunit B	*Methylosarcina fibrata*	
WP_202053530	methane/ammonia monooxygenase subunit B	*Methylomicrobium* sp. RS1	
WP_150047786	methane/ammonia monooxygenase subunit B	*Methylomonas rhizoryzae*	
WP_192392143	methane/ammonia monooxygenase subunit B	*Methylomonas* sp. EbB	
WP_020564880	methane/ammonia monooxygenase subunit C	*Methylosarcina fibrata*	
WP_026223479	methane/ammonia monooxygenase subunit C	*Methylosarcina fibrata*	
**Metaproteomic data**			
WP_014892305	methane monooxygenase	*Methylocystis* sp. SC2	*Hyphomicrobiales (Alphaproteobacteria)*
WP_018408664	MULTISPECIES: methane monooxygenase	*Methylocystis*	
WP_013817027	methane monooxygenase	*Methylomonas methanica*	*Methylococcales (Gammaproteobacteria)*
WP_018408664	methane monooxygenase	*Methylomonas* sp. MK1	
WP_020564882	methane monooxygenase	*Methylosarcina fibrata*	
BAE86886	methane monooxygenase protein B	*Methylomicrobium japanense*	
BAH22841	methane monooxygenase protein C	*Methylomarinum vadi*	
WP_064028764	methane monooxygenase/ammonia monooxygenase subunit B	*Methylomonas koyamae*	
WP_064028760	methane monooxygenase/ammonia monooxygenase subunit C	*Methylomonas koyamae*	
BAJ17642	particulate methane monooxygenase B-subunit	*Methylovulum miyakonense* HT12	
BAJ17640	particulate methane monooxygenase G-subunit	*Methylovulum miyakonense* HT12	
ACE95894	particulate methane monooxygenase subunit A	*Methylomonas methanica*	
WP_010961049	particulate methane monooxygenase subunit alpha	*Methylococcus capsulatus*	

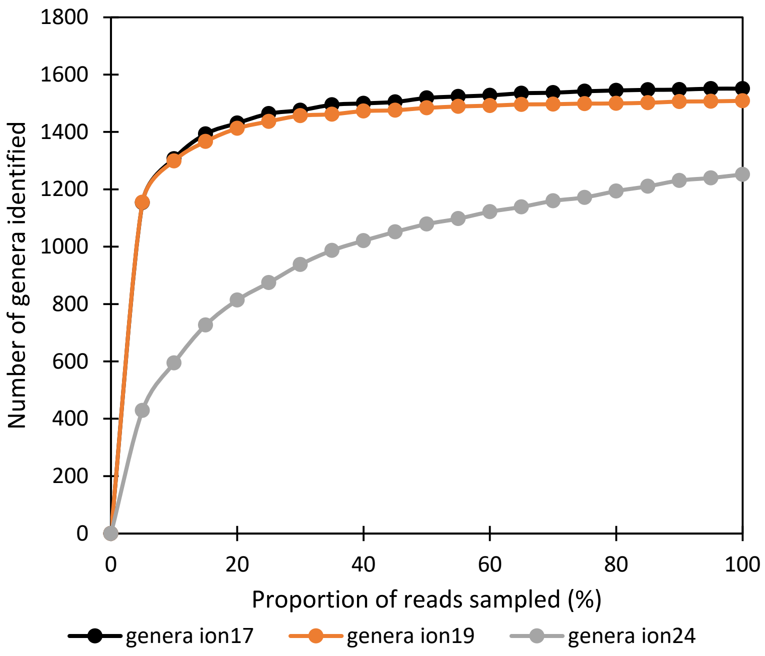

Figure S1. Rarefaction curves representing the relationship between the number of taxa as a function of sequencing depth (proportion of reads sampled). Rarefaction analysis showed that sequencing saturation was achieved almost for all samples.
